# Analysing the Potency of a Seasonal Influenza Vaccine Using Reference Antisera from Heterologous Strains

**DOI:** 10.3390/vaccines12060596

**Published:** 2024-05-30

**Authors:** Christine Wadey, Steven Rockman

**Affiliations:** 1 CSL Seqirus, Melbourne, VIC 3000, Australia; steve.rockman@seqirus.com; 2 Department of Immunology and Microbiology, The University of Melbourne, Parkville, VIC 3010, Australia

**Keywords:** influenza vaccine potency, SRD

## Abstract

The potency of inactivated seasonal influenza vaccine is harmonised by establishing the haemagglutinin (HA) content using the compendial single radial diffusion (SRD) method. SRD reagents (antigens and antisera) are prepared, calibrated and distributed by regulatory agencies as standards for potency testing, following the biannual World Health Organization (WHO) announcements of the virus strains suitable for inclusion in the vaccine. The generation of a homologous hyperimmune sheep antiserum constrains the time to vaccine release. This study tests the application of heterologous antisera to determine the potency of influenza vaccine compared to that of a standard homologous antiserum. The results indicate that the selected heterologous sheep antisera directed to seasonal H1N1, H3N2 or B Victoria virus strains can be used to determine the accurate potency of inactivated seasonal influenza vaccines. Individually selected antisera could be useful for two to fourteen seasons. A limitation to the heterologous antiserum approach is the diversity of each individual serum, indicating that the empirical determination of a specific serum is required. This application has the potential to enable the earlier availability of a seasonal vaccine and reduce animal usage.

## 1. Introduction

Seasonal quadrivalent influenza virus vaccines contain four separate influenza virus subtypes, influenza A (H1N1 and H3N2) and influenza B (B Victoria and B Yamagata). Trivalent vaccines lack the B Yamagata subtype. Due to the continuously changing nature of influenza viruses circulating each season, the WHO makes recommendations for vaccine composition based on the current prevalence, distribution and antigenicity of circulating virus strains for each subtype prior to the southern hemisphere and northern hemisphere vaccine manufacturing seasons. Individual vaccine manufacturers may incorporate alternative strains or reassortants of these strains into the vaccines, provided these have been shown by the two-way Hemagglutination Inhibition (HI) assay to demonstrate a sufficient antigenic match to the recommended vaccine strain. These are thereby listed on the WHO website as suitable candidate vaccine viruses (CVVs) for the season [1,2].

To ensure the effective vaccination of individuals against seasonal influenza, potency is determined for each individual subtype included in the vaccine by measuring the haemagglutinin content using the single radial immunodiffusion (SRD) potency assay [1].

There are alternative approaches to SRD investigated for potency determination, including the antibody-dependent capture detection ELISA, which our group has previously published on [3]. Antibody-independent assays have also been described, such as the surface plasmon resonance assay [4] and reversed-phase high-performance liquid chromatography [5]. While these have been proposed for potency determination, the SRD assay remains the standard which is recognised and accepted by regulatory authorities for potency testing and the release to market of inactivated vaccines. Any alternative potency quantification method would be required to be validated in comparison with SRD before being accepted for vaccine release, and the correlation of this new method with the clinical data and outcomes will need to be confirmed.

The SRD assay based on the method described by Wood et al. [6,7] is used as the standard potency assay currently specified by various pharmacopoeia, such as the United States Pharmacopoeia and British Pharmacopoeia/Pharm Europa. Clinical data are available to support the correlation of SRD potency with vaccine immunogenicity [1,8]. The SRD potency assay is based on the diffusion of viral antigens through an agarose gel matrix containing a specific polyclonal sheep antibody for each individual virus subtype. The globally available antigen standards to be used in the potency assay are calibrated using sodium dodecyl sulphate poly acrylamide gel electrophoresis (SDS-PAGE) or isotopic depletion mass spectroscopy (IDMS) and SRD and distributed with the corresponding antiserum standards by the WHO Essential Reference Laboratories (ERLs): the Therapeutic Goods Administration (TGA), the Medicines and Healthcare products Regulatory Agency (MHRA), the Center for Biologics Evaluation and Research (CBER) and the National Institute of Infectious Diseases (NIID). A defined potency is assigned to each antigen standard. The generation and calibration of antigen and antiserum standards take time, following seasonal WHO notification. It is imperative that the antigen standard matches precisely with the strain of the virus included in the vaccine and to the specific reassortant, if used [1]. It is accepted that the sheep antiserum standard matches to one of the virus strains/clades listed as suitable for use on the WHO website, but is not specifically required to be identical to the reference antigen/vaccine strain [1].

There is a time lag after the seasonal WHO announcement for the generation and calibration of antigen and antiserum standards. The antigen must be generated, dispensed and freeze-dried before calibration. The generation of a specific sheep hyperimmune antiserum has the longest lead time in this process, involving HA purification for the dosing of sheep, the development of an immune response, and then the dispensing of the antiserum. The process may total 10 weeks or more and is not always successful in generating an adequate antibody titre, or in producing single, clear and quantifiable zones in the SRD potency assay [1]. The time to establish, calibrate and distribute these reagents directly impacts vaccine release since antigen potency is required to meet the specification for release of each vaccine lot and is a potential constraint to vaccine production [9].

It has been observed that sheep antisera are often cross-reactive to heterologous viruses from the same or a closely related subtype or clade. It is mandated that the antigen used in SRD assays is matched to the current vaccine strain; however, it would be useful in terms of earlier product availability and for animal ethics considerations if the sheep antiserum generated against viruses produced from vaccine strains from previous seasons or prepared for related strains could be used in the SRD potency assay. This could potentially mean that product availability could be advanced by 4–8 weeks, which would be particularly relevant in the instance of a pandemic. The analysis of the suitability of heterologous antisera for SRD potency assays has been previously investigated for H5N1 vaccine potency determination [8]. Here, we present an investigation of the use of cross-reactive antibodies (heterologous antiserum) as a suitable replacement for a strain-specific (homologous) antiserum in the SRD potency assay for seasonal influenza.

## 2. Materials and Methods

### 2.1. Antigen and Antiserum Standards and Test Samples

Full lists of the antigen and antiserum standards and test samples are shown in Tables S1 and S2 in the Supplementary Materials section.

The antiserum and antigen standards used in this study were prepared by ERLs in collaboration with licensed vaccine manufacturers. Test samples for full potency assays were manufactured drug products suitable for vaccine formulation.

The reference antisera were diluted for use based on the recommendations in the respective Information for Use data sheets supplied by the relevant ERL. The antiserum dilutions used were identical for homologous and heterologous assays. The antigen standards and test samples were diluted to 30 µg HA/mL for the prime dilution based on the ERL assigned potency for antigen standards and for drug substance test samples based on an estimate of HA concentration derived from the tested protein content. The test sample dilutions were modified if required such that the zone diameters fell within those of the standard curve.

### 2.2. Single Radial Diffusion Method

The SRD method was based on the method of Wood et al. [6,7]. Briefly, the influenza antigen samples were disrupted with 10% zwittergent for 30 min at room temperature to solubilise the HA antigen.

In this study, single-point assays were used to evaluate a single dilution of each antigen standard added to a set of four replicate wells on plates which contained either a homologous or heterologous polyclonal sheep antiserum. The degree of cross-reactivity determined by single-point assays was calculated as the percentage difference of the ratio of homologous to heterologous zone size for each antigen.

Percentage difference = 100 × (1 − mean homologous zone diameter/mean heterologous zone diameter).

The level of cross-reactivity was considered as high when the percentage difference between the homologous and heterologous zone sizes was ≤10%. This is based on the observed typical within-assay variability of the SRD assay, with a generally accepted coefficient of variation of ≤15% [8]. Sixty-nine antigen/antiserum pairs were tested.

Based on the results of the single-point assays, full potency assays were performed using either the British Pharmacopoeia/Pharm Europa or United States Pharmacopoeia method for eleven samples of drug substance. The samples were each tested using a homologous antiserum plate and plates each containing one selected heterologous antiserum which had shown cross-reactivity in the single-point assays.

Potency assays tested three dilutions of test sample and homologous antigen standard, each in duplicate to produce dose/response curves for potency calculation. Gels were incubated for 18–24 h at room temperature. Antigen/antibody complexes precipitated as circular or elliptical zones were stained with Coomassie Brilliant Blue. The zone diameter was measured using an ImmuLab image scanner (Parkville, VIC, Australia). Potency titres were calculated using Combistats parallel line bioassay software (Version 6.0) or by regression analysis. Full potency assays were considered to be valid, and results were reported only if all statistical validity criteria were met for both the antigen standard curves and test sample dose/response curves. Studies were not conducted on the cross-reactivity of B Yamagata lineage virus strains as the B/Phuket/3073/2013 strain has been the recommended vaccine strain since 2014.

The percentage difference between homologous and heterologous potency titres was calculated as follows:

Percentage potency difference = 100 × (1 − homologous potency titre/heterologous potency titre).

To evaluate the potential mechanism for the differences observed in antiserum cross-reactivity, an examination was conducted of the degree of homology of the amino acid sequences within the antigenic sites for each pair of virus strains tested within each subtype, as well as the number and comparative positions of potential glycosylation sites. Amino acid sequences were sourced from GISAID [10] and compared using DNASTAR Lasergene version 7 software. Table 1 lists the GISAID isolate identification numbers of the sequences used for strain pair sequence comparison. Positions accepted as antigenic sites were based on the data published by Skowronski et. al. [11] for H1N1 and H3N2 strains and Suptawiwat et. al. [12] for B Victoria strains. Potential N-linked glycosylation sites were identified as a motif of Asparagine-X-Serine/Threonine in the amino acid sequence, where X is any amino acid, excluding proline.

## 3. Results

**Figure 1:**
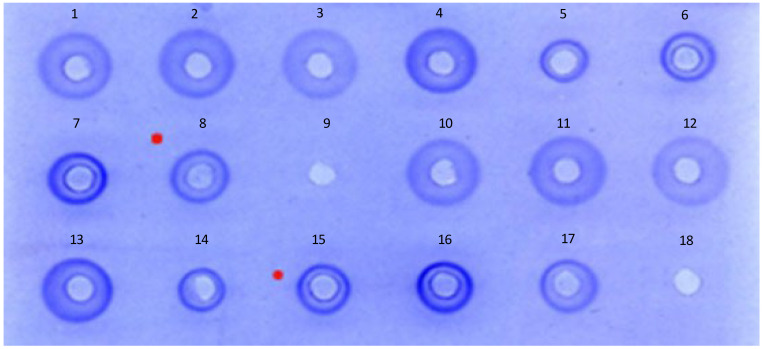
A photographic image of an agarose plate, showing typical examples of homologous and heterologous SRD zones produced by various H3N2 antigen standards and samples on the agarose plate containing A/Darwin/9/2021 antiserum. Wells are assigned numbers from 1–18.

The initial assessment of the degree of cross-reactivity of heterologous antisera showed clear, measurable single precipitation zones for all the homologous and heterologous antigen/antiserum combinations tested. The zone appearance and size were not always consistent between the homologous and heterologous zones. Examples of the zone appearance of both the homologous and heterologous antigens tested with the A/Darwin/9/2021 (H3N2) antiserum are shown in Figure 1.

Antigens:

- Wells 1 and 10: A/Brisbane/1/2018 TGA 2018/125B;
- Wells 2 and 11: A/South Australia/34/2019 TGA 2019/129B;
- Wells 3 and 12; A/Kansas/14/2017 TGA 2019/128B;
- Wells 4 and 13: A/Darwin/6/2021 TGA 2021/138B;
- Wells 5 and 14: A/Cambodia/e0826360/2020 NIBSC 21/100;
- Wells 6 and 15: A/Tasmania/503/2020 Seqirus IVR-221;
- Wells 7 and 16: A/Bangladesh/911009/2020 Seqirus IVR 225;
- Wells 8 and 17: A/Perth/20/2020 Seqirus IVR-220;
- Wells 9 and 18: Blank wells.

To determine the potentially suitable heterologous antiserum/antigen combinations for full SRD potency testing, single-point assays were performed to select the heterologous antisera which produced a zone size correlating sufficiently with the zone size produced by the homologous antiserum.

The degrees of cross-reactivity demonstrated by 69 antigen/antiserum pairs using single-point determinations are shown in Table 2, Table 3 and Table 4. The data represent the zone diameter ratio of a single antigen dilution of the respective antigen standards tested on homologous compared with heterologous antiserum plates.

A positive result for the percentage difference indicates that the heterologous zone size was greater than the corresponding homologous measurement, and a negative result suggests that the homologous zone size was greater than that of the heterologous zone.

The level of cross-reactivity is considered high when the percentage difference between the homologous and heterologous zone sizes is ≤10%. This is highlighted with light grey shading, where it was observed on a heterologous antiserum plate for a more recent virus isolate.

Of the 15 antisera tested for reactivity to fifteen seasonal viruses, 14 showed a high level of cross-reactivity to between one and five heterologous viruses. There were 15 instances of cross-reactivity to viruses which emerged subsequently to the time of isolation of the virus used to generate the sheep antiserum for eight individual antisera (four for H1N1, six for H3N2 and five for B Victoria antisera). For those antisera tested, the B strain antisera appeared to show a higher prevalence of cross-reactivity than the A strains.

**Figure 2:**
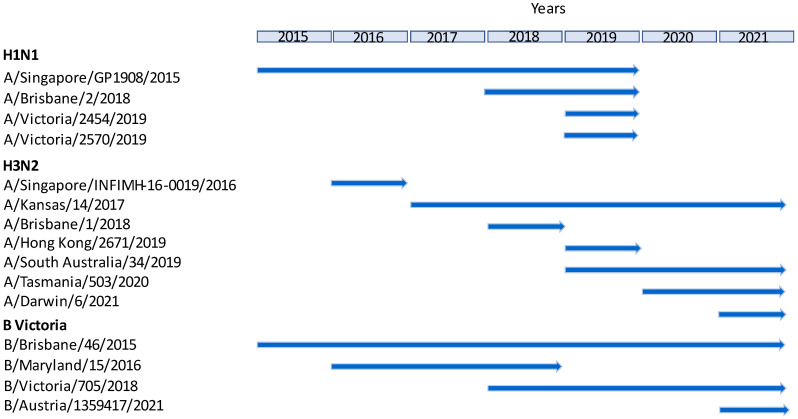
Representation of heterologous antiserum relationships shown as a function of time based on the cross-reactivity data shown in Table 2, Table 3 and Table 4. This representation illustrates as arrows the number of years for which an existing antiserum prepared against a previously circulating strain within a subtype could potentially be a useful alternative to homologous antiserum for the potency assay of a more recently emerging virus strain.

A demonstration of these heterologous relationships as a function of time for each of the antisera tested is shown in Figure 2 and indicates those antisera with a potential application in SRD potency determination. As shown, eight individual antisera demonstrated cross-reactivity to the more recently isolated viruses, spanning between two and fourteen vaccine seasons (two seasons per year). H1N1 antiserum cross-reactivity was shown to carry over for up to 5 years (10 vaccine seasons), that of H3N2 will continue for up to 5 years (10 seasons), and for B Victoria, it was 7 years (14 seasons), which is the longest time period of the three subtypes.

Utilising the sequences from the reference database [10] (see Table 1), the alignment of the amino acid sequence demonstrated the differences between the antigenic sites of the viruses within each subtype. This analysis demonstrated differences of up to 16 amino acids between the pairs of viruses. More specifically, and not unexpectantly, the greatest difference was observed within the antigenic sites of the H3N2 subtype comparison pairs (4–16 differences), compared with H1N1 (5–6 differences) and B Victoria (3–8 differences). The amino acid variation, both overall, and with respect to individual antigenic sites, did not correlate with the level of cross-reactivity determined by the single-point assays. In many cases, the viruses showing the most conserved amino acid composition did not correlate with the most cross-reactive antiserum data, and most diverse amino acid sequences did not result in the lowest cross-reactive antiserum level.

The analysis of the number of potential glycosylation sites for the H1N1 and H3N2 virus strains showed a few differences in the total number of and position of sites, and these differences did not correlate with the differences in cross-reactivity. The newly acquired glycosylation sites did not appear to result in a change in the reactivity pattern. The potential glycosylation sites for B Victoria strains were in conserved positions. Further experimentation would be required to understand the relationship between the amino acid sequence and SRD cross-reactivity, which may relate to the polyclonal nature of the antiserum used.

**Figure 3:**
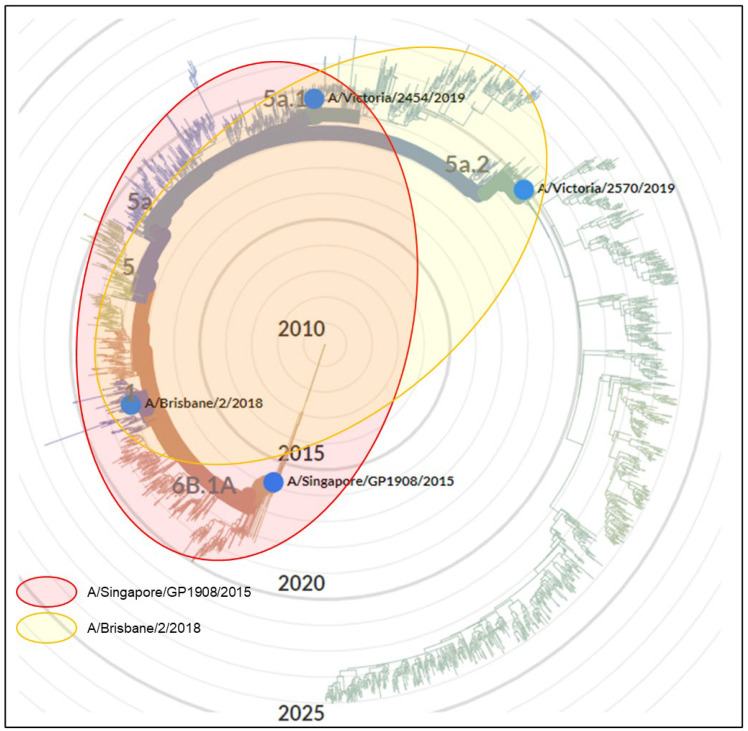
A Venn diagram representation of antiserum cross-reactivity patterns for the H1N1 subtype of virus which emerged subsequently to the virus used to generate the sheep antiserum. Cross-reactivity patterns as determined in Table 2 are indicated by shaded shapes superimposed onto the data derived from the Nextstrain website [13], represented in a radial layout. The original licensed work displayed on the Nextstrain webpage has been modified to include only virus strains tested in the current study. The radial position of the emergence of new antigenic clades/subclades, e.g., clade 6B.1A is indicated as in the Nextstrain webpage.

**Figure 4:**
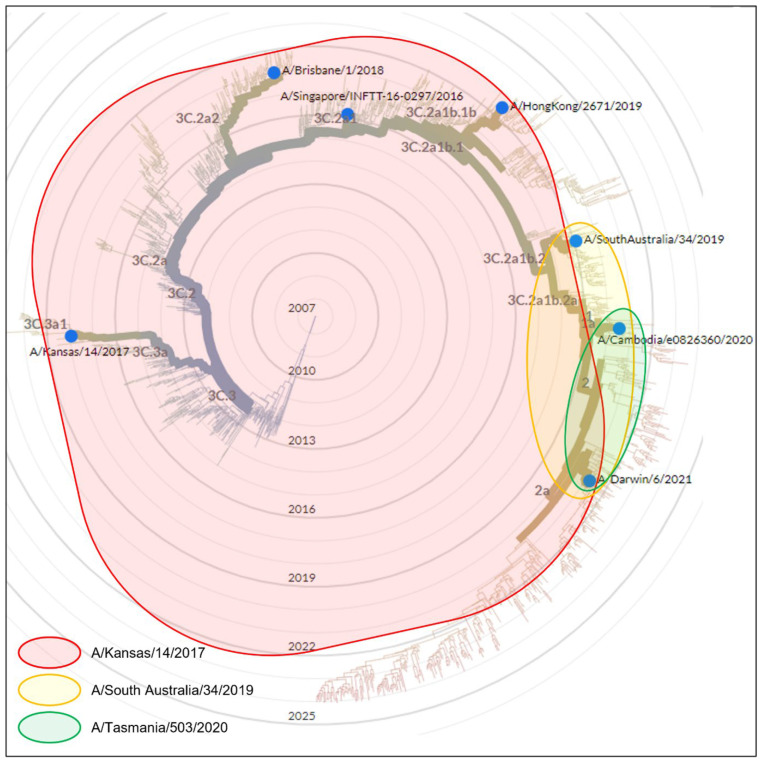
A Venn diagram representation of antiserum cross-reactivity patterns for the H3N2 subtype of virus which emerged subsequently to the virus used to generate the sheep antiserum. Cross-reactivity patterns as determined in Table 3 are indicated by shaded shapes superimposed onto the data derived from the Nextstrain website [13], represented in a radial layout. The original licensed work displayed on the Nextstrain webpage has been modified to include only virus strains tested in the current study. The radial position of the emergence of new antigenic clades/subclades, e.g., clade 2a is indicated as in the Nextstrain webpage.

**Figure 5:**
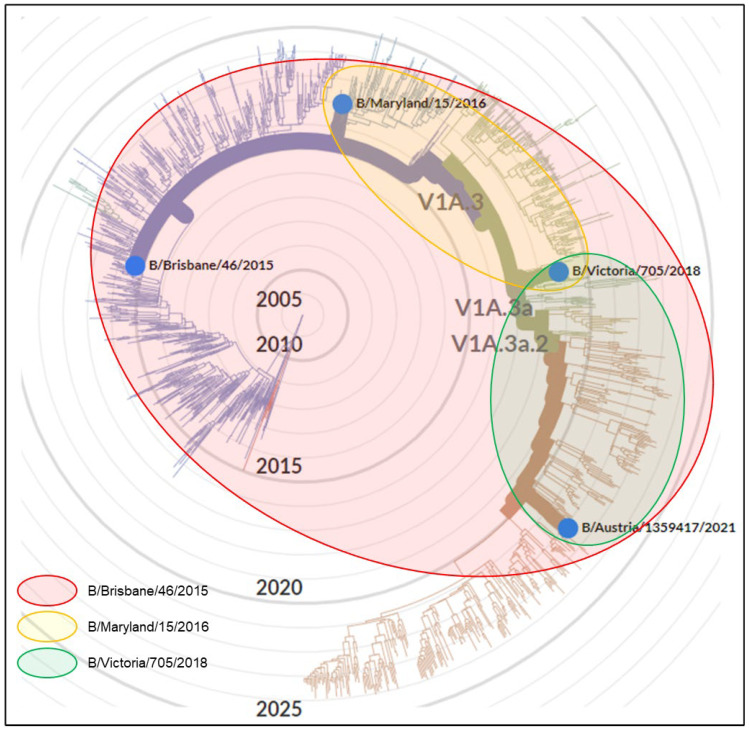
A Venn diagram representation of antiserum cross-reactivity patterns for the B Victoria subtype of virus which emerged subsequently to the virus used to generate the sheep antiserum. Cross-reactivity patterns as determined in Table 4 are indicated by shaded shapes superimposed onto the data derived from the Nextstrain website [13], represented in a radial layout. The original licensed work displayed on the Nextstrain webpage has been modified to include only virus strains tested in the current study. The radial position of the emergence of new antigenic clades/subclades, e.g., clade V1A.3 is indicated as in the Nextstrain webpage.

The antigenic cross-reactivity patterns of antigen and antiserum pairs for the subtypes H1N1, H3N2 and B Victoria derived from Table 2, Table 3 and Table 4 are shown in Figure 3, Figure 4 and Figure 5, respectively, as Venn diagrams of the reactivity patterns superimposed on trees derived from the Nextstrain website [13] and represented in a radial layout. The shaded areas represent the cross-reactivity to the viruses which were isolated at a subsequent time to the isolation of the virus used to generate the sheep antiserum.

Based on the patterns of reactivity observed and the availability of a monovalent drug substance for testing, full potency determinations were performed for eleven commercial drug substance monovalent samples using homologous and selected heterologous antisera. In some instances, more than one heterologous antiserum was evaluated for a given monovalent sample.

The potency results for the drug product test samples are shown in Table 5. A positive result for the percentage difference indicates that the heterologous potency titre was greater than the homologous potency titre, and a negative result suggests that the homologous potency titre was greater than the corresponding heterologous titre.

The potency results using the heterologous antiserum were within 10% of the results obtained using the homologous antiserum for 6/7 H1N1 pairs, 6/6 H3N2 pairs and 5/6 B Victoria pairs. These results indicate that the majority of the heterologous antisera showing cross-reactivity in the single-point assays would potentially be suitable substitutes of homologous antisera for potency testing.

**Figure 6:**
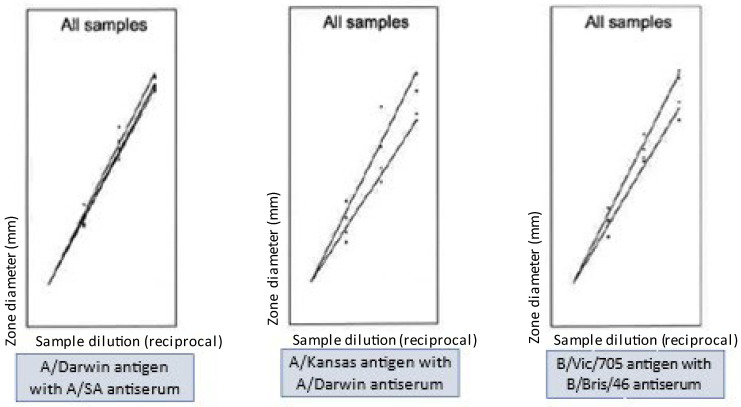
Examples of typical dose/response curves for standard antigen and monovalent drug product on various heterologous antiserum plates. Images are derived from the output of Combistats calculation software.

All the homologous and heterologous potency results listed in Table 5 were determined valid, meeting all statistical validity criteria for both the antigen standard curves and test sample dose/response curves. Some typical examples of the linear dose/response curves on heterologous antiserum plates are shown in Figure 6.

In summary, analysing the data with a forward-looking perspective with respect to the availability of antisera, the changing WHO strain recommendations, and a potency determination of within 10% of the homologous result, the application of an existing heterologous polyclonal antiserum would be feasible for potency determination (H1N1 *n* = 6; H3N2 *n* = 6; B Victoria *n* = 5). A limitation to the heterologous antiserum approach is the diversity of each serum, indicating that the empirical determination of each specific serum would be required.

## 4. Discussion

Due to the constantly evolving nature of influenza virus, the WHO makes updated recommendations for the seasonal influenza vaccine composition based on the prevalence and antigenicity of circulating virus strains for each subtype twice yearly, prior to the southern hemisphere and northern hemisphere vaccine manufacturing seasons [2]. SRD reagents (antigens and antisera) for vaccine potency determination and lot release must be generated with each new recommendation, with a 10–12-week lead time to generate a sheep antiserum in sufficient quantity for global requirements. In some instances, a polyvalent sheep antiserum may produce SRD zones with an indistinct precipitin line, preventing the accurate assessment of the zone diameter. A homologous antiserum may be in limited supply or may be produced too late for the timely release of a vaccine for seasonal immunization programs.

The use of an already available heterologous antiserum with sufficient cross-reactivity may supply a suitable alternative to the generation of a strain-specific antiserum for each WHO-recommended strain change to advance batch release and product availability.

The results of this study support the application of heterologous antisera to determine the potency of an inactivated influenza vaccine as a substitute for a homologous antibody. For all the three subtypes analysed, there were instances of antiserum cross-reactivity to viruses which emerged subsequently to the time of isolation of the virus used to generate the sheep antiserum.

A high level of cross-reactivity was maintained for between 2 and 14 seasons, and for each subtype more than one antiserum could be identified with utility across multiple seasons. The antisera to the B strains in this restricted dataset showed an overall higher level of cross-reactivity and longer potential utility than the A strain antisera analysed, which may be a function of the slower rate of change for the B Victoria lineage reflected by less frequent changes to the WHO strain recommendation than that for the H1N1 and H3N2 lineages [2].

Potency assays comparing the use of a heterologous antiserum for the samples of a commercial monovalent drug substance gave results within 10% of the homologous result for the majority of the antisera tested. From the data presented, it appears that a suitable heterologous antiserum may potentially be available in many cases as an alternative for homologous antiserum.

The utility of this approach is particularly apparent for the H3N2 subtype, which has had the most frequent changes in WHO strain recommendation [2] over the last 10 years. This virus subtype has also demonstrated the most antigenic variation determined by ferret and human serology, as well as changes to the haemagglutinin amino acid sequence [2], according to the WHO bi-annual reports. The patterns of heterologous reactivity demonstrated in this study of seven H3N2 antisera were complex and reflective of the rate of antigenic change in H3N2 virus evolution compared with those of the other subtypes.

The examination of changes to the amino acid sequence within the antigenic sites and superficial patterns of glycosylation were not found to correlate or provide an explanation for the cross-reactivity patterns observed, particularly for the H3N2 virus strains included in this study. It is well understood that glycosylation can affect immunogenicity [14], but more detailed biochemical and structural analysis would be required to draw conclusions regarding the relationship between glycosylation and cross-reactivity. This should encompass the role of the composition of the glycan groups at each glycosylation site.

This study extends on the previous work by Vodeiko and Weir [8], which demonstrated that three available H5N1 heterologous hyperimmune sheep reference antisera prepared to different clades within the H5N1 subtype all showed cross-reactivity to three monovalent vaccine preparations of the same virus strains, as well as showing reactivity to the H1N1 control virus. In contrast, a sheep antiserum to the H1N1 seasonal viruses circulating immediately prior to the emergence of the pandemic A/California/07/2009 H1N1 virus showed no evidence of cross-reactivity to this pandemic strain.

The post-infection ferret antiserum for the virus strains tested using Hemagglutination Inhibition assays was shown by Vodeiko and Weir [8] to be more discriminatory than SRD using a sheep antiserum. These results are consistent with the results of the current study in that the relationships observed for sheep antiserum cross-reactivity did not always mirror the accepted clade structure, which is typically representative of serological relationships determined by ferret and human serology and the viral HA amino acid sequence. The different results for the two methods may also relate to the polyclonal nature of the hyperimmune sheep serum and may reflect the presence of non-specific serum inhibitors in a hyperimmune sheep antiserum.

In summary, analysing the data with a forward-looking perspective with respect to the availability of antisera to meet the changing WHO strain recommendations, the application of an existing heterologous polyclonal antiserum would be feasible for the SRD potency determination of inactivated influenza vaccines. A limitation to the heterologous antiserum approach is the diversity of each serum, indicating that the empirical determination of each specific serum would be required. This study contributes to the published literature on the evaluation of the heterologous antiserum approach for influenza potency determination, which at the time of manuscript preparation, is very limited for both seasonal and pre-pandemic virus strains.

## 5. Conclusions

This study extends on the previous work to demonstrate the potential value of the use of heterologous antiserum standards to determine vaccine potency using SRD. The previous study focused on the applicability of the use of heterologous antibody for potency determination for H5N1 pre-pandemic vaccine, whereas the current study extends this work to include seasonal virus strains. The results demonstrate the value of evaluation of the use of a heterologous antiserum for seasonal influenza vaccine potency determination in the event of a shortage or delay in availability of the homologous antiserum. Application would be subject to the empirical evaluation of each specific serum considered for use, which may involve comparative studies where data are required to assign a conversion factor for potency determination using a heterologous antiserum.

## Figures and Tables

**Table 1:** GISAID Isolate ID references [9] for amino acid sequences used for strain pair comparison.

Subtype	Virus Strain	GISAID Isolate ID
H1N1	A/Brisbane/2/2008	Sequence not available
	A/Singapore/GP1908/2015	EPI-ISL-230287
	A/Victoria/2454/2019	EPI-ISL-413140
	A/Victoria/2570/2019	EPI-ISL-459907
H3N2	A/Singapore/INFIMH-16-0019/2016	EPI-ISL-312185
	A/Kansas/14/2017	EPI-ISL-364380
	A/Brisbane/1/2018	EPI-ISL-314708
	A/Hong Kong/2671/2019	EPI-ISL-414502
	A/South Australia/34/2019	EPI-ISL-391803
	A/Tasmania/503/2020	EPI-ISL-528387
	A/Darwin/9/2021	EPI-ISL-3836436
B/Victoria	B/Brisbane/46/2015	EPI-ISL-197575
	B/Maryland/15/2016	EPI-ISL-282250
	B/Victoria/705/2018	EPI-ISL-397113
	B/Austria/1359417/2021	EPI-ISL-3355461

**Table 2:** H1N1 potency determinations expressed as the percentage difference of the zone diameter ratio of a single antigen dilution of an antigen standard tested on homologous compared with heterologous antiserum plates. Percentage difference = 100 × (1 − mean homologous zone diameter/mean heterologous zone diameter). Grey shading represents antiserum cross-reactivity with a more recently emerging strain.

Percentage Difference	Antiserum	A/Singapore/GP1908/2015	A/Brisbane/2/2018	A/Victoria/2454/2019	A/Victoria/2570/2019
**Antigen**					
A/Singapore/GP1908/2015		0	17	10	31
A/Brisbane/2/2018		−8	0	−2	14
A/Victoria/2454/2019		−1	8	0	4
A/Victoria/2570/2019		−17	−9	−20	0

**Table 3:** H3N2 potency determinations expressed as the percentage difference of the zone diameter ratio of a single antigen dilution of an antigen standard tested on homologous compared with heterologous antiserum plates. Percentage difference = 100 × (1 − mean homologous zone diameter/mean heterologous zone diameter). Grey shading represents antiserum cross-reactivity with a more recently emerging strain.

Percentage Difference	Antiserum	A/Singapore/INFIMH-16-0019/2016	A/Kansas/14/2017	A/Brisbane/1/2018	A/Hong Kong/2671/2019	A/South Australia/34/2019	A/Tasmania/503/2020	A/Darwin/9/2021
**Antigen**								
A/Singapore/INFIMH-16-0019/2016		0	−8	−7	−5	−11	NT *	NT
A/Kansas/14/2017		31	0	12	16	1	−5	2
A/Brisbane/1/2018		25	6	0	16	4	NT	NT
A/Hong Kong/2671/2019		30	9	17	0	3	NT	NT
A/South Australia/34/2019		39	11	19	22	0	1	10
A/Cambodia/e0826360/2020-like		NT	16	NT	NT	9	0	−13
A/Darwin/6/2021		NT	8	NT	NT	7	−1	0

* NT = not tested.

**Table 4:** B Victoria potency determinations expressed as the percentage difference of the zone diameter ratio of a single antigen dilution of an antigen standard tested on homologous compared with heterologous antiserum plates. Percentage difference = 100 × (1 − mean homologous zone diameter/mean heterologous zone diameter). Grey shading represents antiserum cross-reactivity with a more recently emerging strain.

Percentage Difference	Antiserum	B/Brisbane/46/2015	B/Maryland/15/2016	B/Victoria/705/2018	B/Austria/1359417/2021
**Antigen**					
B/Brisbane/46/2015		0	14	4	17
B/Maryland/15/2016		−2	0	−2, −2 *	8
B/Victoria/705/2018		3	7, 9 *	0	11
B/Austria/1359417/2021		0	12	1	0

* two assays performed on separate days.

**Table 5:** Potency determinations used to compare results obtained using homologous and selected heterologous antisera. Percentage difference= 100 × (1 − homologous potency titre/heterologous potency titre). Results in bold did not meet the criterion of ≤10% difference.

Subtype	Test Sample	Heterologous Antiserum	Homologous Potency Titre	Heterologous Potency Titre	Percentage Difference
H1N1	A/Guangdong-Maonan/SWL1536/2019 Lot: 274956	A/Singapore/GP1908/ 2015	831	842	1
	A/Guangdong-Maonan/SWL1536/2019 Lot: 274956	A/Brisbane/2/2018	1037	966	−7
	A/Guangdong-Maonan/SWL1536/2019 Lot: 274956	A/Singapore/GP1908/2015	1025	935	−10
	A/Guangdong-Maonan/SWL1536/2019 Lot: 274956	A/Victoria/2570/2019	1025	999	−3
	A/Victoria/2570/2019 Lot: 294426	A/Victoria/2454/2019	1402	1324	−6
	A/Brisbane/2/2018 Lot: M09061604710	A/Victoria/2454/2019	436	439	1
	A/Singapore/GP1908/2015 Lot: M09061571100	A/Victoria/2454/2019	947	1113	**15**
H3N2	A/Darwin/6/2021 MPH Lot: 340420	A/Kansas/14/2017	1635	1647	1
	A/Darwin/6/2021 MPH Lot Lot: 340420	A/South Australia/34/2019	1635	1752	7
	A/Darwin/6/2021 MPH Lot Lot: 340420	A/Tasmania/503/2020	1304	1327	2
	A/Cambodia/e0826360/2020 Lot: 327660	A/Darwin/9/2021	394	361	−9
	A/Kansas/14/2017 MPH Lot: M09062599620	A/Darwin/9/2021	1375	1259	−9
	A/Kansas/14/2017 MPH Lot M09062599620	A/Tasmania/503/2020	810	862	6
B Victoria	B/Victoria/705/2018 MPH Lot: 316285	B/Brisbane/46/2015	1103	1087	−1
	B/Victoria/705/2018 MPH Lot: 327661	B/Austria/1358417/2021	411	377	−9
	B/Victoria/705/2018 MPH Lot: 327661	B/Brisbane/46/2015	411	408	−1
	B/Maryland/15/2016 Lot: M09063591500	B/Austria/1358417/2021	788	923	**15**
	B/Austria/1358417/2021 Lot: 331022	B/Victoria/705/2018	896	952	6
	B/Austria/1358417/2021 Lot: 331022	B/Brisbane/46/2015	896	980	9

## Data Availability

Raw data is held in electronic format by CSL Seqirus Ltd. and can be made available on request to the authors.

## Supplementary Materials

The following supporting information can be downloaded at: https://www.mdpi.com/article/10.3390/vaccines12060596/s1, Table S1: Influenza virus antigen standards and test samples of commercial drug substance; Table S2: Influenza sheep antiserum standards.
