# Supplementary material for: Analysing the Potency of a Seasonal Influenza Vaccine Using Reference Antisera from Heterologous Strains
Source: Vaccines (Basel). 2024 May 30;12(6):596. doi: 10.3390/vaccines12060596 (PMC11209104; doi:10.3390/vaccines12060596)
Supplement: Supplementary file 1 [file vaccines-12-00596-s001.zip › vaccines-2985539-supplementary.pdf]

vaccines

Supplementary Materials

Table S1. Influenza Viruses antigen standards and test samples of commercial drug substance.

Subtype	Strain	Clade	Supplier and code
H1N1	A/Singapore/GP1908/2015 (IVR-180)	6B.1	TGA 2016/112B
	A/Brisbane/2/2018 (IVR-190)	6B.1A1	TGA 2019/131B
	A/Victoria/2454/2019 (IVR-207)	6B.1A.5a.1	TGA 2020/132B
	A/Victoria/2570/2019 (IVR-215)	6B.1A.5a.2	TGA 2020/134B
	A/Singapore/GP1908/2015<br>Lot: M09061571100	6B.1	Monovalent commercial drug substance
	A/Brisbane/2/2018<br>Lot: M09061604710	6B.1A1	Monovalent commercial drug substance
	A/Guangdong-Maonan/SWL1536/2019<br>Lot: 274956	6B.1A.5a.1	Monovalent commercial drug substance
	A/Victoria/2570/2019<br>Lot: 294426	6B.1A.5a.2	Monovalent commercial drug substance
H3N2	A/Singapore/INFIMH-16-0019/2016 (IVR-186)	3C.2a1	TGA 2018/123B
	A/Kansas/14/2017 (X-327)	3C.3a1	TGA 2019/128B
	A/Brisbane/1/2018 (X-311)	3C.2a2	TGA 2018/125B
	A/Perth/20/2020 (IVR-220)	3C.2a1b.1a	Seqirus Lot 465
	A/Hong Kong/2671/2019 (IVR-208)	3C.2a1b.1b	TGA 2020/133B
	A/South Australia/34/2019 (IVR-197)	3C.2a1b.2	TGA 2019/129B
	A/Bangladesh/911009/2020 (IVR-225)	3C.2a1b.2	Seqirus Lot 515
	A/Cambodia/e0826360/2020 (IVR-224)	3C.2a1b.2a1	NIBSC 21/100

	A/Darwin/6/2021 (IVR-227)	3C.2a1b.2a2	TGA 2021/138B
	A/Kansas/14/2017<br>Lot: M09062599620	3C.3a1	Monovalent commercial drug substance
	A/Cambodia/e0826360/2020<br>Lot: 327660	3C.2a1b.2a1	Monovalent commercial drug substance
	A/Darwin/6/2021<br>Lot: 340420	3C.2a1b.2a2	Monovalent commercial drug substance
B Victoria	B/Brisbane/46/2015	V1A	TGA 2016/111B
	B/Maryland/15/2016	V1A	TGA 2018/122B
	B/Victoria/705/2018 (BVR-11)	V1A.3	TGA 2019/130B
	B/Austria/1359417/2021 (BVR-26)	V1A.3a.2	TGA 2021/139B
	B/Maryland/15/2016<br>Lot: M09063591500	V1A	Monovalent commercial drug substance
	B/Victoria/705/2018<br>Lot: 316285	V1A.3	Monovalent commercial drug substance
	B/Victoria/705/2018<br>Lot: 327661	V1A.3	Monovalent commercial drug substance
	B/Austria/1358417/2021<br>Lot: 331022	V1A.3a.2	Monovalent commercial drug substance

Table S2. Influenza sheep antiserum standards

Subtype	Virus strain used for immunization	Supplier and code
H1N1	A/Brisbane/2/2008	TGA AS430
	A/Singapore/GP1908/2015 (IVR-180)	TGA AS418
	A/Victoria/2454/2019 (IVR-207) suitable for testing of A/Guangdong-Maonan/SWL1536/2019	TGA AS438
	A/Victoria/2570/2019 - like	TGA AS443
H3N2	A/Singapore/INFIMH-16-0019/2016 (IVR-186)	TGA AS424
	A/Kansas/14/2017 (X-327) - like	TGA AS433
	A/Brisbane/1/2018 (X-311)	TGA AS428
	A/Hong Kong/2671/2019 - like	TGA AS439
	A/South Australia/34/2019 - like	TGA AS437
	A/Tasmania/503/2020, suitable for testing of A/Cambodia/e0826360/2020	TGA AS444
	A/Darwin/9/2021 -like	TGA AS445
B/Victoria	B/Brisbane/46/2015	TGA AS411
	B/Maryland/15/2016	TGA AS422
	B/Victoria/705/2018, B/Washington/02/2019 - like	TGA AS436
	B/Austria/1359417/2021	TGA AS446
